# Is intra-articular injection of high-concentration ozone (O_2_–O_3_) more effective in treating knee osteoarthritis? A multicenter cohort study

**DOI:** 10.1097/MD.0000000000051297

**Published:** 2026-10-23

**Authors:** Ridvan Isik, Burak Yildiz, Sena Unver, Savas Sencan, Serdar Kokar, Osman Hakan Gunduz, Emre Uzun, Kemal Nas

**Affiliations:** a Division of Pain Medicine, Department of Physical Medicine and Rehabilitation, Sakarya Training and Research Hospital, Sakarya, Turkey; b Department of Physical Medicine and Rehabilitation, Emet Dr. Fazıl Doğan State Hospital, Kütahya, Turkey; c Division of Pain Medicine, Department of Physical Medicine and Rehabilitation, Sakarya University, Faculty of Medicine, Sakarya, Turkey; d Division of Pain Medicine, Department of Physical Medicine and Rehabilitation, Marmara University, Faculty of Medicine, Istanbul, Turkey; e Department of Physical Medicine and Rehabilitation, Sakarya Training and Research Hospital, Sakarya, Turkey.

**Keywords:** intra-articular injection, knee osteoarthritis, ozone concentration, ozone therapy

## Abstract

Knee osteoarthritis is a prevalent joint disorder globally, resulting in pain and disability, with various treatment options available. Intra-articular ozone (O_2_–O_3_) administrations are an efficient therapeutic approach commonly employed in this condition. With this study, we aimed to compare the effect of varying ozone concentrations on treatment outcomes. This multicenter retrospective cohort study was conducted at 2 distinct university hospitals between May 2023 and September 2024. Medical records of patients who underwent intra-articular knee ozone injections were retrospectively reviewed and divided into 2 groups as 15 μg/mL (group A) and 25 μg/mL ozone (group B). Participants’ pain scores were measured by Numeric Rating Scale, functional status by Western Ontario and McMaster Universities Osteoarthritis Index and quality of life by 12-item Short Form Health Survey before and at 1 and 3 months following ozone therapy. A total of 190 patients were screened; 159 patients (105 females, 54 males) were included in the study, with 77 patients in group A and 82 patients in group B. Both groups demonstrated significant improvements in all outcomes during follow-up. After adjustment for multiple comparisons, between-group differences in Numeric Rating Scale scores were not statistically significant at either 1 or 3 months. Group B had significantly better Western Ontario and McMaster Universities Osteoarthritis Index, 12-item Short Form Health Survey physical component summary, and 12-item Short Form Health Survey mental component summary scores at both follow-up assessments (all Holm-adjusted *P* < .001). The 25 μg/mL concentration was associated with more favorable short-term functional and quality-of-life outcomes and a trend toward greater pain improvement. These findings should be considered exploratory and require confirmation in prospective randomized studies.

**Key Points**

- Intra-articular ozone injections are an efficient treatment modality in patients with knee osteoarthritis.
- High-concentration ozone therapy elicited more favorable results.
- For severe osteoarthritis, high-concentration ozone may be an alternative option.

## 1. Introduction

Osteoarthritis (OA) is the most common form of arthritis. Its prevalence continues to increase in parallel with the aging global population, posing a significant threat to public health.^[1]^ The mechanism of OA entails a reduction in cartilage tissue and structural abnormalities within the joint area. As a result of these impairments, patients suffer from symptoms such as severe pain, joint stiffness, and swelling, as well as limited motion.^[2]^

The primary objective of treatment for patients with knee osteoarthritis (KOA) is to alleviate pain and enhance daily activities. Treatment methods for relieving pain and improving daily performance encompass analgesics, nonsteroidal anti-inflammatory drugs, physiotherapy, injections, and surgical interventions. A variety of injection methods such as corticosteroids, platelet-rich plasma, ozone, and hyaluronic acid are administered into the joint in patients who are not suitable for surgical treatment and refractory to conservative treatments.^[3–5]^

Ozone (O_2_–O_3_) therapy is widely used in clinical practice as an alternative medical approach in the treatment of musculoskeletal diseases. Ozone reduces tissue damage by neutralizing reactive oxygen species and promotes the activation of antioxidant mechanisms.^[6]^ The actions of ozone therapy on OA are based on its interaction with reactive oxygen species and modulation of cellular mechanisms in cartilage tissue.^[7,8]^ Many recent studies have reported that intra-articular ozone implementation has a positive impact on KOA.^[9–11]^ Most available studies have focused on the comparison of ozone therapy with other intra-articular injections. However, the influence of the concentration and/or volume of the ozone used during injection on treatment outcomes has been omitted. The concentration of ozone administered into the joint varies, typically ranging from 15 to 30 μg/mL in these studies; however, there is no consensus regarding the appropriate dosage to be used. Despite the widespread use of intra-articular ozone, no clinical study has directly compared different ozone concentrations using standardized ultrasound (US)-guided injections in a multicenter cohort.

Based on this gap in the literature, we aimed to compare pain, functional status, and quality of life among patients treated with different concentrations of intra-articular ozone therapy. Furthermore, establishing the optimal dosage of intra-articular ozone therapy, commonly employed in OA treatment, will provide valuable guidance for clinicians.

## 2. Materials and methods

### 2.1. Research design and population

This multicenter retrospective cohort study was carried out between May 2023 and September 2024 in 2 university hospital outpatient pain clinics. After obtaining our university ethics committee approval (approval number: E-71522473-050.04-372963-174). Following ethics committee approval, the electronic medical records of patients who had undergone intra-articular ozone injection for KOA during the study period were retrospectively reviewed. Treatment exposure had occurred as part of routine clinical practice before the initiation of the retrospective data analysis; therefore, no prospective allocation or methodological blinding was performed. This study was conducted in accordance with the principles of the Declaration of Helsinki. This study is reported in accordance with the Strengthening the Reporting of Observational Studies in Epidemiology guidelines. Written informed consent was obtained from all patients before the procedure.

In this study, demographic data, symptom duration, injected ozone concentration and volume, number of knees injected (uni/bilateral), and presence of any complications in the postinjection period were collected by examining the hospital registration system. The injected knees of all patients were evaluated based on the Kellgren–Lawrence (KL) grading system. We recruited patients with at least grade II according to the KL classification system.

#### 2.1.1. Inclusion criteria

Patients aged 50 to 85 years with a diagnosis of primary KOA according to the American College of Rheumatology^[12]^ criteria, symptomatic knee with KL^[13]^ grades II, III, and IV, who underwent intra-articular ozone treatment.

#### 2.1.2. Exclusion criteria

Patients with a history of any intra-articular injection within 3 months before the first ozone injection, a history of previous knee surgery, a diagnosis of malignancy, missing follow-up parameters, bilateral intra-articular knee ozone injection, a secondary KOA diagnosis (trauma, inflammatory joint disease, etc), patients without demographic and clinical data, or direct radiographs.

### 2.2. Groups

Both ozone concentrations were administered at each participating center during the study period. Treatment concentration was selected as part of routine clinical practice, without randomization or a prespecified study allocation protocol. Patients were retrospectively categorized according to the ozone concentration documented in their medical records: 15 μg/mL (group A) or 25 μg/mL (group B). Patients in group A received 15 μg/mL to 10 mL and patients in group B received 25 μg/mL to 10 mL ozone intra-articular injection. Due to the short half-life of ozone (about 45 minutes at 20°C), we produced it fresh using an ozone generator (TURKOZONE Blue-S – Turkey) connected to a pure oxygen source and used it immediately for the patient. Ozone generators use oxygen through high-voltage tubes in the output range of 4000 to 14,000 and produce an ozone-oxygen mixture with concentration ranges up to 5%.

A total of 3 sessions of intra-articular injections were performed in symptomatic knees of all patients, once a week. We provided the patients with stretching and strengthening exercises of quadriceps, hamstring, and hip group muscles in the form of a home exercise program, 3 sets and 10 repetitions per day until the first month control after injection.

The primary outcome measure of the study was the change in the numerical rating scale for pain following intervention. Secondary outcome measures were changes in Western Ontario and McMaster Universities Osteoarthritis Index (WOMAC) and 12-item Short Form Health Survey (SF-12) scores and presence of complications.

### 2.3. Injection procedure

In both centers, the procedures were performed in a special injection room under US guidance (HM70 EVO, Samsung Healthcare) by qualified pain medicine physicians (RI and SS). Patients underwent standard monitoring throughout the procedure, including electrocardiography, noninvasive blood pressure, and peripheral oxygen saturation, and an intravenous catheter was placed pre-procedure as a precaution.

The linear US probe (10–12 MHz) was enclosed in a sterile cover, and then the patient’s skin was sterilized 3 times with iodopovidone. A customized sterile gel was used as the sonographic interface. Patients were asked to lie supine, and then an US probe was initially placed in a longitudinal plane (long axis) above the distal femur. The US probe was slightly shifted towards the patella apex, and once the suprapatellar recess image was attained, the probe was rotated to the transverse plane (short axis). A 22-gauge, 80-mm needle (Stimuplex A; BBraun) was advanced from lateral to medial using an in-plane approach to insert the tip into the suprapatellar recess. After confirming negative aspiration, a 10 mL ozone (O_2_–O_3_) injection (group A: 15 µg/mL, group B: 25 µg/mL) was delivered to both groups.

Patients were followed up in the observation room for 30 minutes after the injection for the presence of any possible complication. Patients with no complications were discharged with recommendations and asked to return for follow-up visits after 1 and 3 months.

### 2.4. Outcome measures

We evaluated the data of pain, functional status, and quality of life scales (Numeric Rating Scale [NRS], WOMAC, and SF-12, respectively) in the hospital registry system of patients who had the procedure. The NRS, WOMAC, and SF-12 scales were employed prior to the procedure and at 1 and 3 months post-procedure.

WOMAC: This questionnaire was developed to assess joint function associated with OA and consists of 3 main sections: pain, stiffness, and physical function. Turkish validity and reliability study was conducted.^[14]^
SF-12: This short form aims to measure the physical and mental health status of the patients and includes a total of 12 questions. A Turkish validity and reliability study was made.^[15]^
NRS (0–11): It is a widely used self-report tool to assess patients’ pain levels.^[16]^

### 2.5. Sample-size considerations

Because of the retrospective design, no a priori sample-size calculation was performed. The study sample comprised all eligible patients identified from the available medical records during the predefined study period who had complete outcome data.

### 2.6. Statistical analysis

Descriptive values of the measurements were calculated as mean, standard deviation, median, 25th and 75th percentiles, number, and % frequencies depending on the way the characteristics were attained. The compliance of the numerical measurements with the normal distribution was examined by the Shapiro–Wilk test, and it was determined that age and body mass index measurements conformed to the normal distribution, but the other scale scores did not show normal distribution. Independent-samples *t* test was used to compare the 2 groups in terms of normally distributed characteristics. Mann–Whitney *U* test was used to compare 2 groups in terms of numerical measurements that did not show normal distribution, and the Friedman test was used to compare 3 measurement periods. Significantly different periods were determined by post hoc Dunn test. The relationships between categorical variables were analyzed by Pearson chi-square test. *P* < .05 was accepted as statistical significance level, and all statistical analyses were performed using IBM SPSS Statistics, version 23.0 (IBM Corp.).

As a supplementary analysis to address potential confounding associated with the nonrandomized allocation between the 2 ozone-concentration groups (15 and 25 μg/mL), propensity score weighting was performed. Propensity scores were estimated using a logistic regression model including age, sex, body mass index, occupation, marital status, KL grade, and baseline NRS, WOMAC, 12-item Short Form Health Survey physical component summary (PCS-12), and 12-item Short Form Health Survey mental component summary (MCS-12) scores. Stabilized inverse probability of treatment weights were then calculated for participants in both treatment groups. Covariate balance before and after weighting was assessed using standardized mean differences (SMDs), with an absolute SMD of <0.10 considered indicative of acceptable balance. Weighted mean changes from baseline were calculated for each outcome, and adjusted between-group differences were expressed as group B − group A. For NRS and WOMAC, positive changes represented score reductions and clinical improvement, whereas for PCS-12 and MCS-12, positive changes represented score increases and clinical improvement. Confidence intervals (CIs) were estimated using 1000 stratified bootstrap samples, with the propensity score model reestimated in each sample. Holm method was used to adjust *P* values for the 8 weighted outcome comparisons.

Since NRS, WOMAC, PCS-12, and MCS-12 scores were not normally distributed, these outcomes were summarized as medians and interquartile ranges. Between-group comparisons were performed using the Mann–Whitney *U* test, and rank-biserial correlation was calculated as a nonparametric effect-size measure. The magnitude of rank-biserial correlation was interpreted as small (|*r*| < 0.30), moderate (|*r*| = 0.30–0.49), or large (|*r*| ≥ 0.50).

To account for multiple testing, Holm method was used to control the family-wise Type I error rate. Between-group *P* values were adjusted across the 12 outcome-by-time comparisons. For each significant Friedman test, *P* values from the 3 post hoc pairwise comparisons were Holm-adjusted within each outcome and treatment group. *P* values from the 8 propensity score–weighted outcome comparisons were also adjusted using Holm method. All tests were two-sided, and a Holm-adjusted *P* value of <.05 was considered statistically significant.

Clinical significance analyses were additionally performed using responder thresholds derived from previously published studies. Substantial clinical improvement for pain reduction was defined as at least a 50% reduction in NRS scores from baseline values.^[17]^ For WOMAC scores, clinically meaningful improvement was defined as a decrease of at least 10 points from baseline.^[18]^ For the SF-12 physical and mental component summary scores, clinically meaningful improvement was defined as an increase of at least 4 points in PCS-12 and MCS-12 scores from baseline values.^[19]^

## 3. Results

A total of 190 patients were screened; 159 patients with knee conditions (105 females, 54 males) were included in the study, and data of each patient’s symptomatic knee were analyzed (Fig. 1). The mean age of the participants was 63.06 ± 7.03 (40–81) years, and the mean duration of symptoms was 25.59 ± 16.55 (6–72) months. The distribution of the patients in terms of age, gender, occupation, marital status, symptom duration, and KL grade is given in Table 1. The examination of the table reveals a similar distribution of demographic and clinical conditions between the 2 groups.

**Table 1:** Demographic and clinical characteristics of the study groups.

Characteristic	Category	Group A (15 μg/mL; n = 77)	Group B (25 μg/mL; n = 82)	*P* value
Age (yr)		63.49 ± 6.19	62.66 ± 7.74	.456
BMI (kg/m^2^)		29.17 ± 2.62	29.83 ± 2.98	.145
Sex	Female	50 (64.9)	55 (67.1)	.907
	Male	27 (35.1)	27 (32.9)	
Symptom duration (mo)		24.80 ± 15.80	26.43 ± 17.38	.538
Occupation	Homemaker	31 (40.3)	40 (48.8)	.063
	Civil servant	19 (24.7)	9 (11.0)	
	Worker	14 (18.2)	9 (11.0)	
	Retired	9 (11.7)	16 (19.5)	
	Unemployed	4 (5.2)	8 (9.8)	
Marital status	Married	67 (87.0)	75 (91.5)	.523
	Single	6 (7.8)	3 (3.7)	
	Widowed	4 (5.2)	4 (4.9)	
KL grade	Grade 2	46 (59.7)	44 (53.7)	.649
	Grade 3	26 (33.8)	30 (36.6)	
	Grade 4	5 (6.5)	8 (9.8)	

Data are presented as mean ± standard deviation or n (%), as appropriate. Continuous variables were compared using the independent-samples *t* test, and categorical variables using Pearson chi-square test.

BMI = body mass index, KL = Kellgren–Lawrence.

**Figure 1.**
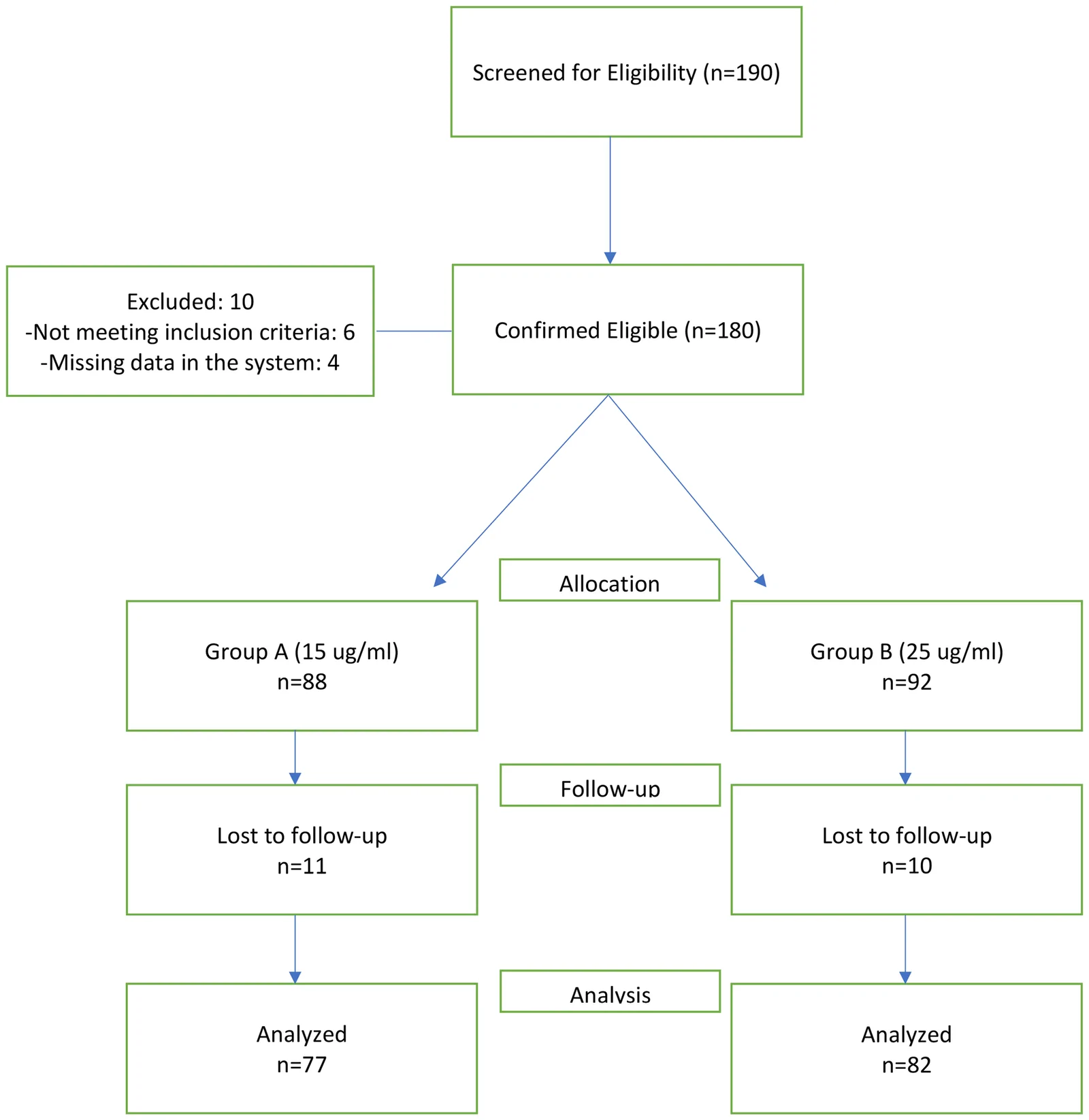
Flowchart.

The results of the intergroup comparisons in each measurement period and the intragroup comparisons (between measurement periods) in the 2 groups in terms of NRS, WOMAC, and SF-12 scores are presented in Table 2. When the table is examined:

**Table 2:** Clinical outcome measures at baseline, 1-month, and 3-month follow-ups, with nonparametric effect-size analysis.

Outcome	Assessment	Group A (15 μg/mL; n = 77) Median (IQR)	Group B (25 μg/mL; n = 82) Median (IQR)	Holm-adjusted *P* value*	Rank-biserial correlation *rᵣ*ᵦ	Within-group *P* value† Group A	Within-group *P* value† Group B
NRS	Baseline	8.00 (7.00–9.00)	8.00 (7.00–8.00)	1.000	0.022	<.001	<.001
	1 month	4.00 (2.00–5.00)	3.00 (2.00–4.00)	.293	0.172		
	3 months	5.00 (3.00–6.00)	4.00 (3.00–5.00)	.088	0.221		
WOMAC	Baseline	76.00 (64.00–83.00)	72.50 (61.00–82.00)	.798	0.118	<.001	<.001
	1 month	41.00 (31.00–58.00)	30.50 (21.25–40.00)	<.001	0.374		
	3 months	57.00 (42.00–69.00)	39.50 (31.00–51.00)	<.001	0.456		
PCS-12	Baseline	30.23 (26.42–33.99)	30.14 (25.75–33.95)	1.000	0.025	<.001	<.001
	1 month	38.43 (33.92–40.66)	41.55 (38.37–44.64)	<.001	0.384		
	3 months	34.76 (30.62–38.26)	38.23 (35.75–41.47)	<.001	0.430		
MCS-12	Baseline	33.67 (30.76–37.45)	34.78 (32.23–37.47)	.915	0.094	<.001	<.001
	1 month	40.41 (38.56–43.21)	43.79 (41.76–47.65)	<.001	0.432		
	3 months	37.67 (34.56–40.31)	40.61 (37.80–44.11)	<.001	0.458		

Data are presented as median (interquartile range).

IQR = interquartile range, MCS-12 = 12-item Short Form Health Survey mental component summary, NRS = Numeric Rating Scale, PCS-12 = 12-item Short Form Health Survey physical component summary, WOMAC = Western Ontario and McMaster Universities Osteoarthritis Index.

* Between-group comparisons were performed using the Mann–Whitney *U* test; *P* values were adjusted across the 12 outcome-by-time comparisons using Holm method. Rank-biserial correlation was calculated as the nonparametric between-group effect-size measure and is presented as an absolute value; values <0.30, 0.30–0.49, and ≥0.50 were interpreted as small, moderate, and large effects, respectively. Effect direction should be interpreted together with the group medians because lower scores indicate improvement for NRS and WOMAC, whereas higher scores indicate improvement for PCS-12 and MCS-12.

† Within-group comparisons were performed using the Friedman test. All Holm-adjusted post hoc pairwise comparisons (baseline vs 1 month, baseline vs 3 months, and 1 month vs 3 months) were *P* < .001 in both groups.

- Median NRS scores decreased significantly from baseline at both follow-up assessments in both groups (all Holm-adjusted post hoc *P* < .001). Between-group differences were not statistically significant at either 1 month (Holm-adjusted *P* = .293; |*rᵣ*ᵦ|=0.172) or 3 months (Holm-adjusted *P* = .088; |*rᵣ*ᵦ|=0.221). Although group B had numerically lower median NRS scores at both follow-up assessments, the corresponding effect sizes were small.
- WOMAC, PCS-12, and MCS-12 scores improved significantly from baseline at both follow-up assessments in both groups (all Holm-adjusted post hoc *P* < .001). At both 1 and 3 months, group B had significantly lower median WOMAC scores and higher median PCS-12 and MCS-12 scores than group A (all Holm-adjusted *P* < .001). The corresponding rank-biserial correlations indicated moderate between-group effects (|*rᵣ*ᵦ|=0.374–0.458).

Changes in median NRS and WOMAC scores across the assessment periods are presented in Figure 2, with error bars representing the interquartile ranges.

**Figure 2.**
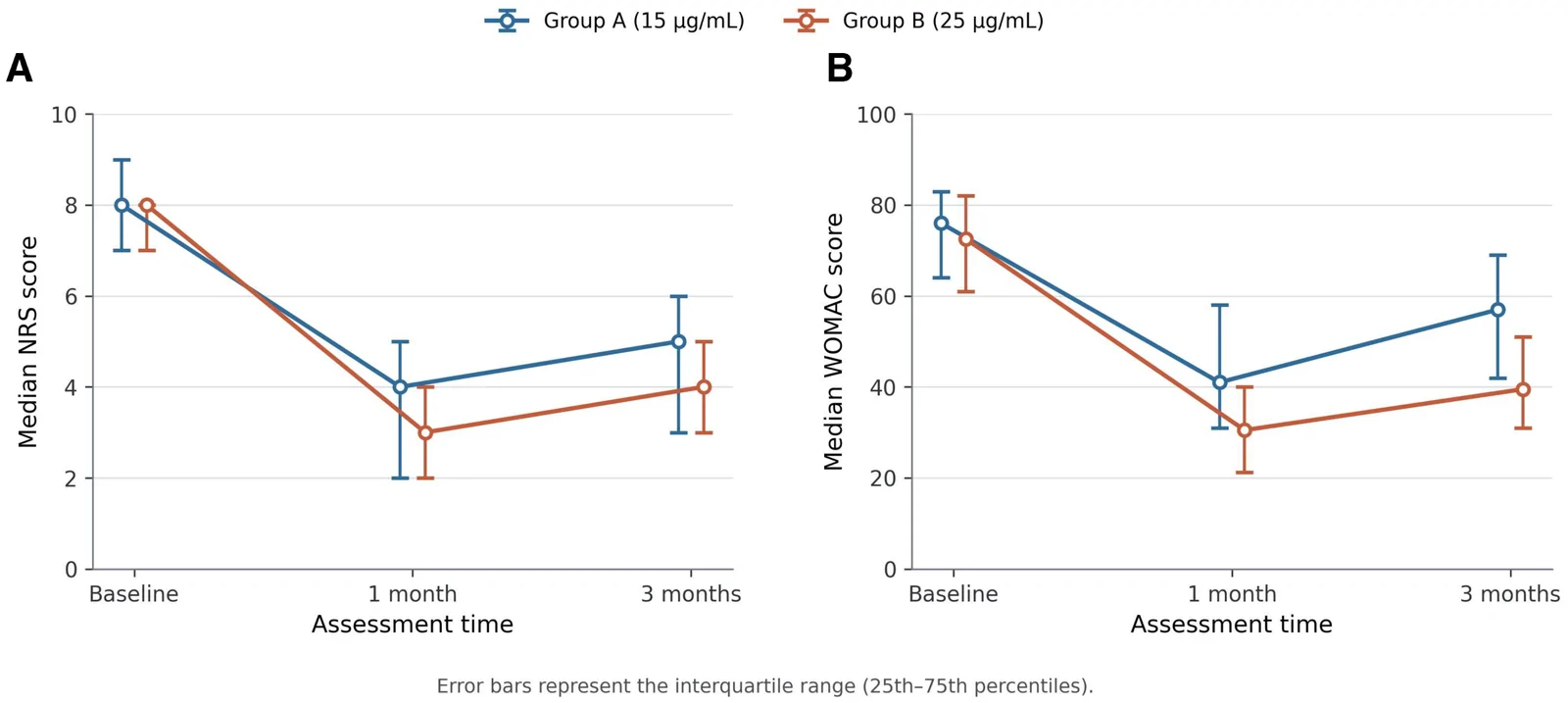
The changes in median NRS and WOMAC scores from baseline to 1- and 3-month follow-ups in the two treatment groups. (A) Changes in median NRS scores. (B) Changes in median WOMAC scores. Error bars represent the interquartile ranges (25th–75th percentiles). NRS = Numeric Rating Scale; WOMAC = Western Ontario and McMaster Universities Osteoarthritis Index.

The comparison of NRS between groups in each measurement period of patients according to KL grading is given in Table 3. After Holm adjustment across the 9 KL-grade-by-assessment comparisons, none of the between-group differences reached statistical significance, although a borderline difference favoring group B was observed among patients with KL grade 3 at 3 months (Holm-adjusted *P* = .054).

**Table 3:** The comparison of NRS between groups in each measurement period of patients according to KL grading.

KL grade	NRS	Groups	n	25	Median	75	Holm-adjusted *P* value*
2	NRSPT	Group A	46	6.75	8.0	9.0	1.000
		Group B	44	7.0	8.0	9.0	
	NRS1m	Group A	46	2.0	3.0	4.0	1.000
		Group B	44	1.0	2.0	3.0	
	Nrs3m	Group A	46	3.0	4.0	5.0	.288
		Group B	44	2.0	3.0	4.0	
3	NRSPT	Group A	26	7.0	8.0	9.0	1.000
		Group B	30	7.0	8.0	8.25	
	NRS1m	Group A	26	4.0	5.0	5.25	1.000
		Group B	30	3.0	4.0	5.0	
	NRS3m	Group A	26	5.0	6.0	7.0	.054
		Group B	30	4.0	5.0	6.0	
4	NRSPT	Group A	5	7.5	8.0	8.0	1.000
		Group B	8	6.5	8.0	8.75	
	NRS1m	Group A	5	5.5	6.0	6.0	1.000
		Group B	8	4.25	5.5	6.0	
	NRS3m	Group A	5	7.0	7.0	8.0	.315
		Group B	8	6.0	6.5	7.0	

1m = 1 month, 3m = 3 months, KL = Kellgren–Lawrence, NRS = Numeric Rating Scale, PT = pretreatment.

* Between-group comparisons were performed using the Mann–Whitney *U* test. *P* values were adjusted across the 9 KL-grade-by-assessment comparisons using Holm method.

### 3.1. Effect size and clinical significance analysis

At baseline, between-group effect sizes were negligible or small for all outcome measures (|*rᵣ*ᵦ|=0.022–0.118), indicating generally comparable baseline distributions. At 1 month, the between-group effect was small for NRS (|*rᵣ*ᵦ|=0.172) and moderate for WOMAC, PCS-12, and MCS-12 (|*rᵣ*ᵦ|=0.374, 0.384, and 0.432, respectively). Similarly, at 3 months, the effect remained small for NRS (|*rᵣ*ᵦ|=0.221), whereas moderate effects were observed for WOMAC, PCS-12, and MCS-12 (|*rᵣ*ᵦ|=0.456, 0.430, and 0.458, respectively). These findings indicate that the between-group differences were more pronounced for functional status and health-related quality of life than for pain intensity (Table 2).

Clinical significance analysis showed that 31 of 77 patients (40.3%) in the group A and 47 of 82 patients (57.3%) in the group B achieved substantial clinical improvement, defined as a ≥50% reduction in NRS scores from baseline. The proportion of patients achieving substantial pain reduction was significantly higher in the group B than the group A (χ^2^ = 4.62, *P* = .032; Table 4 and Fig. 3).

**Table 4:** Clinical significance analysis based on NRS, WOMAC, PCS-12, and MCS-12 responder criteria at 3-month follow-up.

Outcome	Group	Responder, n (%)*	Nonresponder, n (%)	Holm-adjusted *P* value†	Chi-square (χ^2^)
NRS	Group A (15 μg/mL)	31 (40.3%)	46 (59.7%)	.063	4.62
	Group B (25 μg/mL)	47 (57.3%)	35 (42.7%)		
WOMAC	Group A (15 μg/mL)	60 (77.9%)	17 (22.1%)	.063	4.55
	Group B (25 μg/mL)	74 (90.2%)	8 (9.8%)		
PCS-12	Group A (15 μg/mL)	30 (39.0%)	47 (61.0%)	<.001	30.50
	Group B (25 μg/mL)	67 (81.7%)	15 (18.3%)		
MCS-12	Group A (15 μg/mL)	31 (40.3%)	46 (59.7%)	<.001	27.01
	Group B (25 μg/mL)	66 (80.5%)	16 (19.5%)		

MCS-12 = 12-item Short Form Health Survey mental component summary, NRS = Numeric Rating Scale, PCS-12 = 12-item Short Form Health Survey physical component summary, WOMAC = Western Ontario and McMaster Universities Osteoarthritis Index.

* Responders were defined as patients achieving a ≥50% reduction in NRS score, a ≥10-point reduction in WOMAC score, and a ≥5-point improvement in PCS-12 or MCS-12 score from baseline.

† Between-group comparisons were performed using Pearson chi-square test; *P* values were adjusted across the 4 responder-outcome comparisons using Holm method.

**Figure 3.**
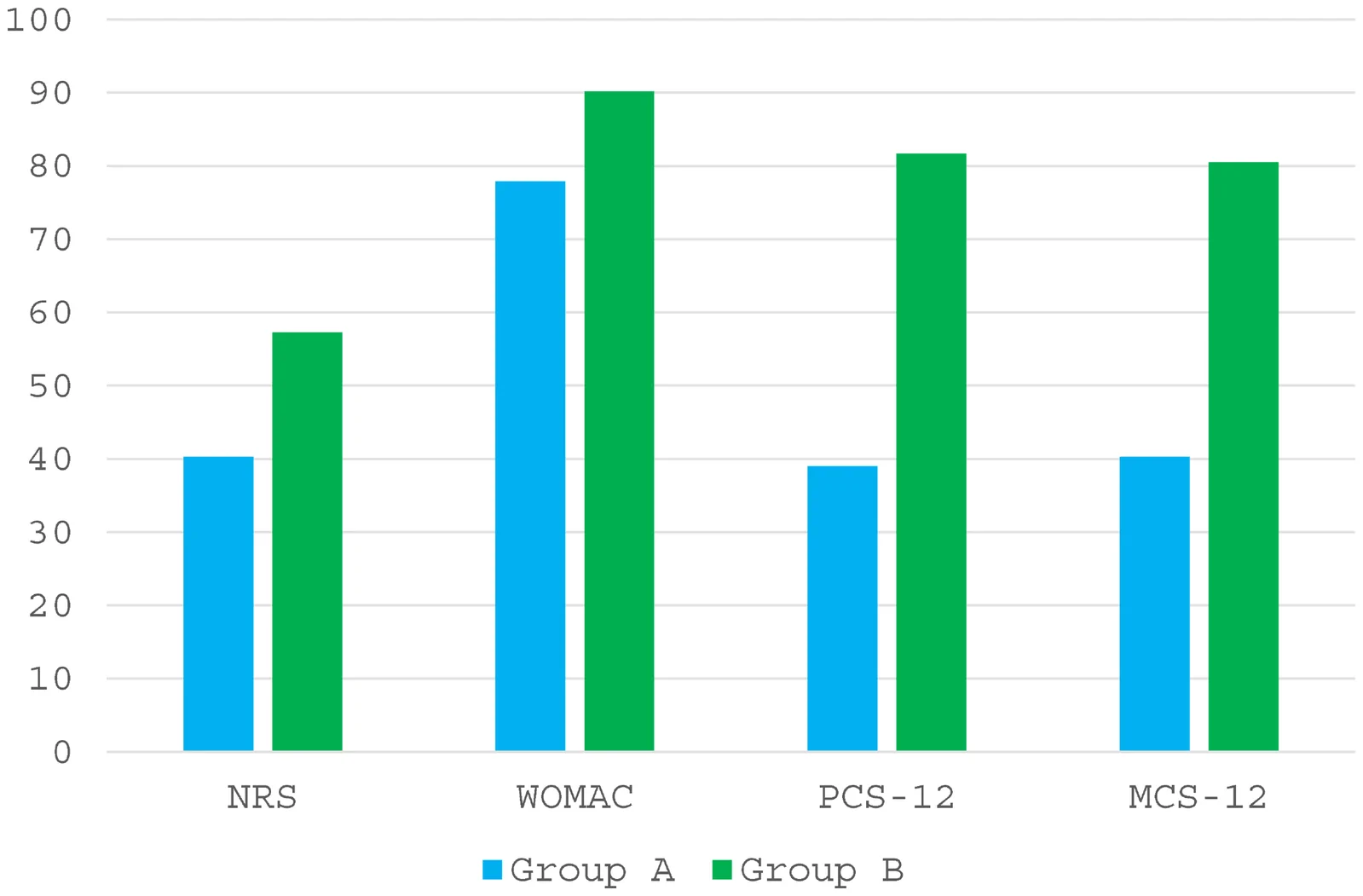
Responder rates according to NRS, WOMAC, PCS-12, and MCS-12 criteria at 3-month follow-up. MCS-12 = 12-item Short Form Health Survey mental component summary, NRS = Numeric Rating Scale, PCS-12 = 12-item Short Form Health Survey physical component summary, WOMAC = Western Ontario and McMaster Universities Osteoarthritis Index.

Clinical significance analysis for WOMAC scores showed that 60 of 77 patients (77.9%) in the group A and 74 of 82 patients (90.2%) in the group B achieved clinically meaningful improvement, defined as a ≥10-point reduction in WOMAC scores from baseline. The proportion of patients achieving clinically meaningful WOMAC improvement was substantially higher in the group B than the group A (χ^2^ = 4.55, *P* = .033; Table 4 and Fig. 3).

Clinical significance analysis for SF-12 component scores showed that PCS-12 responder rates were 39.0% in the group A and 81.7% in the group B. The proportion of PCS-12 responders was notably higher in the group B (χ^2^ = 30.50, *P* < .001). Similarly, MCS-12 responder rates were 40.3% in the group A and 80.5% in group B, with a significantly higher responder rate in the group B (χ^2^ = 27.01, *P* < .001; Table 4 and Fig. 3).

### 3.2. Propensity score–weighted analysis

Propensity score weighting achieved adequate balance across all measured baseline covariates. The maximum absolute SMD decreased from 0.364 before weighting to 0.060 after weighting, and no covariate had an absolute SMD of ≥0.10 after weighting. Stabilized weights ranged from 0.57 to 2.64, with no evidence of extreme weights. The weighted effective sample size was 139.6.

In the weighted analysis, the adjusted between-group difference in NRS improvement at 1 month did not reach statistical significance (adjusted mean difference = 0.49; 95% CI = −0.01 to 1.00; Holm-adjusted *P* = .054). At 3 months, group B demonstrated a greater adjusted improvement in NRS than group A (adjusted mean difference = 0.75; 95% CI = 0.40–1.15; Holm-adjusted *P* = .008).

Group B also demonstrated greater adjusted improvements in WOMAC, PCS-12, and MCS-12 scores at both follow-up assessments. At 3 months, the adjusted between-group differences were 12.83 points for WOMAC (95% CI = 10.08–16.52), 4.19 points for PCS-12 (95% CI = 2.86–5.47), and 2.85 points for MCS-12 (95% CI = 1.90–3.65); all Holm-adjusted *P* values were .008. The complete propensity score–weighted outcome results are presented in Table S1, Supplemental Digital Content 1.

No serious complications were observed in both groups except for transient pain at the procedure site in 1 patient in group B and temporary nausea in 1 patient in group A.

## 4. Discussion

In this multicenter retrospective cohort study, both 15 and 25 μg/mL intra-articular ozone injections were associated with clinically relevant improvements in pain, function, and health-related quality of life in patients with KOA. However, the higher ozone concentration produced consistently greater gains in functional status and quality of life and a more pronounced reduction in pain at 3 months.

Our propensity score–weighted analysis supports these observations by showing that the association between the higher concentration and better outcomes persisted after balancing measured baseline characteristics. Although the nonrandomized design prevents causal inference, the consistency between unadjusted and weighted results strengthens the plausibility that ozone concentration contributes meaningfully to the magnitude of clinical benefit. Nevertheless, because treatment allocation was not randomized, the findings should not be interpreted as demonstrating a causal effect of ozone concentration.

Pain is the most common symptom of KOA, and the main goal of treatment in patients is to reduce pain and maximize daily performance. Ozone injection therapy for individuals with KOA has shown efficacy in alleviating knee pain when compared to placebo.^[20,21]^ Pain reduction was observed in both groups; however, the partial rebound in NRS scores by the third month suggests that the symptomatic benefit may diminish over time. The lower 3-month pain scores observed in group B may indicate a more sustained short-term response with the higher concentration. We assume that exercise therapy given to all patients as a home program also contributed importantly to these results. Although the home exercise program may have contributed to the observed improvements, adherence was not objectively monitored; therefore, its influence on the outcomes could not be determined.

The KL grading system (grades 0–IV) for KOA has been utilized by the majority of systematic reviews when examining reviews in this field. Most of the studies have focused on patients in the early or intermediate stages of KOA (KL II and III).^[22]^ The inclusion of patients with KL grade IV disease extends the available evidence to a population that has been underrepresented in previous studies. Although the findings suggest that concentration may influence clinical response in advanced disease, they are insufficient to recommend routine use of a higher concentration without confirmation in randomized trials.

The primary drawback of amplifying the efficacy with greater concentration was that a minor percentage of patients (~1%) experienced pain during injection; nevertheless, this was transient and necessitated no further intervention. This finding, different from other trials, may serve as a catalyst for future research that may reflect the need for increased concentration to enhance the clinical response as the disease advances.

The available data on the optimal dosage of ozone therapy for KOA is scarce. In an animal study conducted by Chen et al, the impact of intra-articular ozone injections on synovial tumor necrosis factor alpha levels was evaluated, revealing a decrease in tumor necrosis factor alpha levels with escalating doses.^[23]^ In another study comparing ozone concentrations, higher doses similarly resulted in greater oxygenation, along with enhanced anti-inflammatory and antioxidant effects.^[24]^ These experimental findings provide a biologically plausible explanation for the observed between-group differences, potentially through concentration-dependent modulation of inflammatory and oxidative pathways; however, these mechanisms were not directly evaluated in our study.

As the severity of OA in individuals with KOA increases, the functional state of the knee diminishes, resulting in noticeable disabilities in physical activities such as walking, stair climbing, and squatting. In a recent systematic review and meta-analysis carried out by Rahimzadeh et al,^[25]^ 12 studies investigating the efficacy of platelet-rich plasma and ozone injections on the WOMAC score in KOA were examined. This meta-analysis concluded that ozone injection is an effective short-term therapy method, consistent with the current study. The physical symptoms of KOA have a direct impact on other aspects of the patient’s life, such as social interactions, mental functioning, and sleep quality. Thus, health-related quality of life measures are relevant and key additional outcomes that aid in gauging the physical, social, and emotional impact of KOA and various OA treatments.^[26]^ According to a recent comprehensive review, OA has a substantial and major impact on quality of life.^[27]^ A meta-analysis by Oliviero et al, encompassing 6 randomized controlled trials and 353 patients with KOA, investigated the efficacy of ozone injection for degenerative knee disease. The authors established that ozone therapy is a promising option that can enhance joint function and patients’ quality of life.^[28]^ The concurrent improvements in pain, physical function, and health-related quality of life observed in group B suggest that the higher ozone concentration may be associated with broader short-term patient-perceived benefits. However, given the nonrandomized design and limited follow-up, these findings should not be interpreted as evidence of a causal or sustained superiority of the higher concentration.

Research on the safety of ozone therapy stated transient mild side effects in certain studies; nevertheless, adverse effects were rarely reported, leading to the notion that the procedure is safe.^[29,30]^ The mild and transient nature of the observed adverse events is consistent with the favorable tolerability profile reported in the literature, although definitive conclusions regarding safety cannot be drawn.

In recent years, there has been a growing interest in the impact of ozone on KOA, with a corresponding rise in the number of studies conducted in this field. Yet, the optimal therapy protocol regarding ozone concentration, injection volume, frequency of injections, and intervals between them remains unclear. For this reason, to the best of our knowledge, our research is valuable, as it is the first study in the extant literature to compare different concentrations. Moreover, the multicenter design, large patient cohort, inclusion of individuals with advanced KOA (KL-IV), and US-guided injections are additional features that strengthen the value of our study.

This study has several limitations. First, its retrospective, nonrandomized design is susceptible to selection bias and confounding by indication. Although propensity score weighting achieved adequate balance across the measured baseline characteristics, it could not account for unmeasured patient-level factors or potential center-related differences; therefore, residual confounding cannot be excluded, and the observed associations should not be interpreted as evidence of an independent causal concentration–response effect. Furthermore, the retrospective design precluded an a priori sample-size calculation, and the study sample was determined by the number of eligible patients with complete outcome data available during the predefined study period. Second, the follow-up period was limited to 3 months, preventing assessment of the long-term durability of the observed clinical improvements. Third, adherence to the prescribed home exercise program was not objectively monitored, and potential differences in adherence between the groups may have influenced the outcomes. Finally, although the multicenter design may improve generalizability, variations in clinical practice between the participating centers cannot be entirely excluded.

## 5. Conclusion

Both ozone concentrations were associated with short-term improvements in pain, function, and quality of life in patients with KOA. Although greater improvements in some outcomes were observed with 25 μg/mL ozone, the retrospective, nonrandomized design precludes causal conclusions regarding concentration-related superiority. Further randomized studies with longer follow-up are required to determine parameters such as optimum concentration, frequency of injection, and total number of sessions, as well as to establish standardized protocols.

## Author contributions

**Conceptualization:** Ridvan Isik.

**Data curation:** Ridvan Isik, Burak Yildiz, Sena Unver.

**Formal analysis:** Ridvan Isik, Savas Sencan, Emre Uzun.

**Methodology:** Ridvan Isik, Burak Yildiz, Savas Sencan, Serdar Kokar, Osman Hakan Gunduz, Emre Uzun.

**Investigation:** Sena Unver, Savas Sencan, Serdar Kokar.

**Resources:** Sena Unver, Osman Hakan Gunduz.

**Visualization:** Sena Unver.

**Supervision:** Serdar Kokar, Osman Hakan Gunduz, Kemal Nas.

**Writing – original draft:** Ridvan Isik, Burak Yildiz.

**Writing – review & editing:** Savas Sencan, Serdar Kokar, Emre Uzun, Kemal Nas.


