# Supplementary material for: Is intra-articular injection of high-concentration ozone (O2–O3) more effective in treating knee osteoarthritis? A multicenter cohort study
Source: Medicine (Baltimore). 2026 Oct 23;105(43):e51297. doi: 10.1097/MD.0000000000051297 (PMC13706393; doi:10.1097/MD.0000000000051297)
Supplement: Supplementary file 1 [file medi-105-e51297-s001.docx]

**Supplementary Table S1. Propensity score–weighted comparisons of changes in clinical outcomes between the treatment groups**

| **Outcome** | **Follow-up** | **Weighted mean improvement Group A (15 μg/mL)** | **Weighted mean improvement Group B (25 μg/mL)** | **Adjusted difference (B − A)** | **Bootstrap 95% CI** | **Holm-adjusted p value** |
| --- | --- | --- | --- | --- | --- | --- |
| **NRS** | 1 month | 4.21 | 4.71 | 0.49 | −0.01 to 1.00 | 0.054 |
| **NRS** | 3 months | 3.06 | 3.81 | 0.75 | 0.40 to 1.15 | 0.008 |
| **WOMAC** | 1 month | 29.46 | 39.66 | 10.21 | 7.35 to 13.60 | 0.008 |
| **WOMAC** | 3 months | 16.63 | 29.46 | 12.83 | 10.08 to 16.52 | 0.008 |
| **PCS-12** | 1 month | 7.93 | 11.56 | 3.63 | 2.56 to 4.73 | 0.008 |
| **PCS-12** | 3 months | 4.31 | 8.50 | 4.19 | 2.86 to 5.47 | 0.008 |
| **MCS-12** | 1 month | 7.26 | 9.97 | 2.71 | 1.76 to 3.67 | 0.008 |
| **MCS-12** | 3 months | 3.71 | 6.57 | 2.85 | 1.90 to 3.65 | 0.008 |

**Note.** Values are propensity score–weighted mean changes from baseline. For NRS and WOMAC, positive values indicate score reduction and clinical improvement; for PCS-12 and MCS-12, positive values indicate score increase and clinical improvement. The propensity score model included age, sex, body mass index, occupation, marital status, Kellgren–Lawrence grade, and baseline NRS, WOMAC, PCS-12, and MCS-12 scores. Stabilized inverse probability of treatment weights were used. Confidence intervals were estimated using 1,000 stratified bootstrap samples, with the propensity score model re-estimated in each sample. P values were adjusted for eight comparisons using Holm’s method. CI, confidence interval; NRS, Numeric Rating Scale; WOMAC, Western Ontario and McMaster Universities Osteoarthritis Index; PCS-12, Physical Component Summary; MCS-12, Mental Component Summary.
